# Relationship Between Personality Traits and Perceived Pain After Photorefractive Keratectomy: A Cross-Sectional Study in Hamadan

**DOI:** 10.31661/gmj.v13i.2989

**Published:** 2024-03-10

**Authors:** Noushin Bazzazi, Mohammad Ahmadpanah, Mostafa Badri, Mohammad Aliseifrabie, Mehdi Alizade

**Affiliations:** ^1^ Department of Ophthalmology, Hamadan University of Medical Sciences, Hamadan, Iran; ^2^ Research Center for Behavioral Disorders and Substances Abuse, Hamadan University of Medical Sciences, Hamadan, Iran; ^3^ Hamadan University of Medical Sciences, Hamadan, Iran; ^4^ Department of Social Medicine, Hamadan University of Medical Sciences, Hamadan, Iran

**Keywords:** Personality Traits, Pain, Photorefractive Keratectomy, NEO Five-Factor Inventory

## Abstract

Background: Photorefractive keratectomy (PRK) is a common procedure to correct refractive errors. However, postoperative pain is one of the most common drawbacks of PRK. Evidence shows that individual`s personality traits could impact postoperative perceived pain. Hence, this study aimed to investigate the severity of postoperative pain and personality traits after PRK.Materials and Methods: This cross-sectional study was performed on 300 patients who attended to Mahdieh Surgical Clinic (Hamadan, Iran) and underwent PRK. Persian version of the NEO Five-Factor Inventory (NEO-FFI) was applied to assess personality traits, and postoperative pain was measured using the visual analog scale (VAS) score.Results: The mean age of patients was 31.8 ±5.51 years, and most (68%) were female. The most common personality trait was neuroticism, and the mean refractive errors of the right and left eyes of the patients were 1.2±2.67 and 1.3±2.75, respectively. Regarding the VAS score, patients with neuroticism traits perceived the most severe postoperative pain (VAS=6±2.2). Also, the Pearson correlation test indicated a positive correlation between perceived pain and neuroticism personality traits (r=0.059, P˂0.001). In contrast, a significant negative correlation was observed between extraversion and conscientiousness with pain perception (r= -0.737 and r= -0.307, respectively).Conclusion: Our findings showed a positive and significant correlation between three personality traits and pain perception in these patients. Postoperative pain perception in patients undergoing PRK depends on the personality traits of these patients. Groups with less personality stability feel more pain than groups with stable personalities.

## Introduction

Photorefractive keratectomy (PRK) was first performed on the human eye in 1988 as a standard, effective, and safe method to treat refractive errors, including myopia and myopic astigmatism [1]. This procedure involves removing the corneal epithelium before the examiner laser [1][2]. Nevertheless, this surgical technique is gaining popularity because it diminishes the risk of ectasia and eliminates flap complications that could occur after laser in situ keratomileusis (LASIK). Indeed, PRK may allow for safer vision correction in a broader range of patients, including some with thinner corneas and/or mild topographic abnormality [3][4]. However, acute postoperative pain was reported as the common side effect of PRK due to the removal of corneal epithelium during the procedure [5]. The main cause of pain seems to be heat damage caused by the Excimer laser [6]. In patients with high refractive errors, the corneal temperature sometimes could increase to 50-60 °C [7]. Although pain from the corneal origin is very severe and stressful for patients and their surgeons, there is no ideal or universally accepted pain control strategy [5]. Hence, many pro-LASIK surgeons consider postoperative pain as the only reason for the refused PRK.

Despite knowing the physical causes and origin of pain, personality traits may be considered as another factor that affects pain intensity, which patients perceive after surgery [8]. Evidence revealed that the type and severity of individuals’ reactions to stress are not always directly related to the severity of stressors but could primarily reflect they perceive the event and/or the degree of sense of danger and threat [8]. Actually, the perception of a potentially stressful event depends on personality traits as an intermediate and intervening variable [8].

The International Association for the Study of Pain (IASP) defines pain as an unpleasant feeling and an emotional experience that is caused by real or possible tissue damage and/or could be explained in the form of such damage [9]. In the experience of pain, tissue damage, and unpleasantness are considered as sensory and affective dimensions, respectively [10]. In fact, in the definition of pain, emotional and cognitive factors play a significant role in explaining this experience [11]. Pain is a mental experience, and because it has unpleasant sensory and affective components, it can coincide with any symptoms and emotional disorders-especially mood and anxiety disorders [12]. Anxiety, depression, and anger are important factors that could increase pain perception [13]; however, positive emotions usually decrease perceived pain [13]. Also, cognitive factors, including attention, expectancy, and appraisal could either increase or decrease pain experiences depending on their specific focus and content [13][14]. Previous studies investigated the role of personality in the variability of pain perception and introduced the Five-Factor Model (FFM) theory of personality [15][16]. According to FFM theory, various behaviors and a comprehensive set of traits could be attributed and generalized into five domains of personality, including neuroticism, extraversion, openness, agreeableness, and conscientiousness [16]. Although the FFM has been used to evaluate associations between personality and pain (both in clinical and experimental pain settings), the findings have been inconsistent [17]. Hence, this study aimed to evaluate the correlation between perceived pain after PRK and personality traits.

## Materials and Methods

Patients

This descriptive cross-sectional study was performed on 300 patients who were candidates for PRK that attended to Mahdieh Surgical Clinic of Hamadan, Iran, from 2020 to 2022. All the patients underwent PRK by the same surgeon. Briefly, patients were anesthetized with anestocaine drop (Sina Darou, Iran), and after ablation, the ocular surface was rinsed with a cold balanced salt solution, and then a bandage contact lens was placed on the cornea [18]. Also, medications were administered, including ofloxacin (one drop daily for one week), ketorolac (one drop for the first 24 hours, then every 12 hours), and betamethasone (one drop every six hours) for one month.

Sample Size Calculations

Regarding the Gadde et al. study [19], considering a significance level (α) of 0.05, a statistical power of 0.8, and an effect size of 0.5, the final sample size was calculated as 250 patients.

Inclusion and Exclusion Criteria

All the patients aged 18 to 80 years were enrolled in the study. Also, patients with corneal pathologies (such as ocular surface disease, keratoconus, a history of ocular herpetic disease), history of previous refractive surgery, presence of systemic disease, pregnancy, lactation, moderate to severe dry eye, current and/or history of psychiatric illness, received any systemic or topical non-steroidal anti-inflammatory drugs, and myopia intensity greater than six diopters were excluded from the study.

Data Collection

The complete ocular examinations, including determination of corrected and uncorrected visual acuity, examination with the slit lamp, Goldman tonometry, indirect ophthalmoscopy, manifest and cyclo-refraction, and imaging with Pentacam (Oculus GmBH, Wetzlar, Germany) were performed by an experienced ophthalmologist. The personality assessment was performed using the Persian version of the NEO Five-Factor Inventory (NEO-FFI) [20]. The NEO-FFI consists of 60 questions (12 questions for each domain), and five major domains of personality (i.e., neuroticism, extroversion, agreeableness, openness, and conscientiousness) were rated with a five-Likert scale (from 0= completely disagree to 4= completely agree). Also, pain assessments were done 24 hours after surgery with a visual analog scale (VAS) using horizontal lines 10 cm long numbered from zero to ten, where zero indicates no pain, and ten was considered as the worst imaginable pain. At the one-week visit, patients were also asked to specify the day they felt the most pain during the past week.

Ethical Considerations

This study was approved by the Research Ethics Committees of Hamadan University of Medical Sciences (approval code: IR.UMSHA.REC.1398.508). Also, written informed consent was obtained from all the patients.

Statistical Analysis

Data were presented as mean and standard deviation (SD) or frequency and percent. All the analyses were performed using IBM SPSS Statistics for Windows, version 21 (IBM Corp., Armonk, NY., USA). Also, Chi-square, ANOVA, and Pearson correlation tests were applied.

## Results

**Table:** Tabale1. Frequency of Personality Traits Based On NEO-FFI

**Personality traits **	**Number**	**Percent**		**NEO-FFI score **	
			Minimum	Maximum	Mean±SD
**Neuroticism**	8	2.6	31	60	43.75±6.77
**Extraversion**	37	12.4	15	55	35.9±6.38
**Openness**	25	8.4	12	32	24.05±5.72
**Agreeableness**	48	16	12	50	41.64±5.52
**Conscientiousness**	182	60.6	28	50	28.16±6.51

**Table:** Table2. The Age and Gender of Studied Patients Based On Personality Traits.

**Variables**	**Neuroticism**	**Extraversion**	**Openness**	**Agreeableness**	**Conscientiousness**	**P-value**
**Gender, n(%) **						
Male	8 (100)	11 (29.7)	8 (15.3)	13 (27)	56 (30.7)	0.43*
Female	0 (0)	26 (70.3)	17 (84.7)	35 (73)	126 (69.3)	
**Age, y (mean±SD) **	26±4.24	30.45±4.92	26.75±7.12	30.46±6.87	31.13±5.92	0.18**

^*^ Chi-square test ^**^ANOVA test

The mean age of total patients was 31.8 ±5.51 years (ranged 18 to 40 years), and 204 (68%) were female. The mean refractive errors (spherical equivalent) in the right and left eyes among these patients were 1.2±2.67 and 1.3±2.75, respectively. Also, the mean VAS score of total patients was 5.37±2.9, and the most postoperative pain was experienced on the first day after surgery. Based on NEO-FFI, the most common personality trait among studied patients was conscientiousness (Table-1).

As shown in Table-2, the mean age of patients was not statistically different among the personality traits (P=0.18). Also, there were no significant differences in the term gender according to personality traits (P=0.43, Table-2).

Regarding the severity of postoperative pain, the mean VAS score of patients was significantly different among personality traits (P=0.039, Table-3).

The Pearson correlation coefficient test revealed a positive and significant correlation between perceived pain and neuroticism personality traits (r=0.059, P˂0.001, Table-4). Moreover, as indicated in Table-4, a negative and significant correlation was observed between extraversion and conscientiousness with pain perception (r= -0.737 and r= -0.307, respectively).

## Discussion

Our findings indicated significant differences among studied patients regarding postoperative pain perception based on personality traits. Although there was a positive correlation between neuroticism and perception of postoperative pain, a negative correlation was observed in patients with extraversion and conscientiousness personality traits.

Despite the efficacy and safety of PRK, postoperative pain remains a severe and potential limitation for accepting this procedure. In PRK, a direct injury of the nerves of the treated cornea occurs, and pain is associated with the healing process [21][22][23].

Individuals’ perception of a potentially stressful event depends on personality traits. Indeed, differences in personality traits are one of the most important causes of stress and perception of a stressful situation [8][10][13]. The role of personality traits on behavior and cognition is sometimes direct and sometimes mediated by affecting factors, e.g., coping patterns, which are a set of behavioral and cognitive processes to prevent, manage, and reduce stress [8]. Also, postoperative pain after PRK could be associated with the patients’ age, pain threshold, motivation, and psychological condition [8]. Numerous studies have focused on the relationship between age, gender, operation time, and surgery platform on postoperative pain perception, but in most of them, no significant association was observed [24][25]. Our study revealed no significant differences between pain intensity with age and gender. However, Moradi-Farsani et al. showed that pain intensity in the first 45 minutes after cataract surgery was higher in women and elderly patients [26].

Kadu et al. [27] indicated that attitude, personality traits, and pain perception have a definite role in patient cooperation and the success of orthodontic treatments. In line with our study, patients with the conscientiousness personality trait experienced lower pain.

Our findings indicated a negative correlation between extraversion and pain perception. In other words, a high level of extroversion is associated with increased positive affect, a large amount of happiness and health, and a positive acceptance of pain [28]. In contrast, severe pain perception among our studied patients was observed in the neuroticism group.

Regarding previous studies, individuals with anxiety have a more negative prediction of events and estimate the negative aspects of events, such as uncontrollability and predictability [29][30]. Consequently, they are prone to negative experiences such as fear, sadness, and anger, and these negative emotions increase their perception of pain [30]. Therefore, neuroticism has a direct and high relationship with pain perception.

The small sample size and unmeasured other patient characteristics (e.g., marital status, educational level, occupational status, etc.) with possible effects on personality traits as well as pain perception were the most important limitations of our study.

## Conclusion

**Table:** Table3. The Severity of Postoperative Pain Among Studied Patients Based On Personality Traits

Personality traits	VAS score		P-value
	Mean±SD	95%CI	
Neuroticism	6±2.2	3-9	
Extraversion	4±2.68	2.4-6	
Openness	3.87±2.27	1.2-6.8	0.039*
Agreeableness	4.93±2.01	3.4-6.9	
Conscientiousness	4.35±1.71	3.7-5.1	

**CI:** Confidence interval; **VAS:**Visual analogue scale; ^*^ANOVA test

**Table:** Table4. Correlation of VAS Score and Personality Traits

Personality traits	r*	P-value
Neuroticism	0.059	˂0.001
Extraversion	-0.737	˂0.001
Openness	-0.125	0.216
Agreeableness	-0.049	0.26
Conscientiousness	-0.307	˂0.001

^*^ Correlation coefficient

The present study indicated that postoperative pain perception among patients undergoing PRK depended on personality traits. Individuals with less personality stability (such as anxious personalities) feel more pain than groups with stable personalities. Neuroticism was related to the high level of anxiety and pain perception; however, extroversion and more interaction with others led to low sensitivity and reduction of extreme focus on stimuli related to pain, depression, and helplessness.

## Conflicts of Interests

The authors of the study declare there were no conflicts of interest.

## References

[R1] Vestergaard AH, Hjortdal J, Ivarsen A, Work K, Grauslund J, Sjølie AK (2013). Long-term outcomes of photorefractive keratectomy for low to high myopia:
13 to 19 years of follow-up. J Refract Surg.

[R2] Spadea L, Verboschi F, De Rosa, Salomone M, Vingolo EM (2015). Long-term results of no-alcohol laser epithelial keratomileusis and
photorefractive keratectomy for myopia. Int J Ophthalmol.

[R3] Moisseiev E, Sela T, Minkev L (2013). Increased preferences of surface ablation over LASIK between 2008-2011 is
correlated to risk of ectasia. Clin Ophthalmol.

[R4] Guedj M, Saad A, Audureau E (2013). PRK in patients with suspected KCN; five years follow up. J Cataract Refract Surg.

[R5] Garcia R, Campello Horovitz, Torricelli A (2016). Improved Evaluation of postoperative Pain after PRK. Cornea.

[R6] Ishihara M, Arai T, Sato S, Morimoto Y, Obara M, Kikuchi M (2001). Temperature measurement for energy-efficient ablation by thermal
radiation with a microsecond time constant from the corneal surface during ArF
excimer laser ablation. Front Med Biol Eng.

[R7] Maldonado-Codina C, Morgan PB, Efron N (2001). Thermal consequences of photorefractive keratectomy. Cornea.

[R8] Shimizutani M, Odagiri Y, Ohya Y, Shimomitsu T, Kristensen TS, Maruta T, et al (2008). Relationship of nurse burnout with personality characteristics and coping
behaviors. Ind Health.

[R9] Treede RD (2018). The International Association for the Study of Pain definition of pain:
as valid in 2018 as in 1979, but in need of regularly updated footnotes. Pain Rep.

[R10] Hofbauer RK, Rainville P, Duncan GH, Bushnell MC (2001). Cortical representation of the sensory dimension of pain. J Neurophysiol.

[R11] Rainville P, Feine JS, Bushnell MC, Duncan GH (1992). A psychophysical comparison of sensory and affective responses to four
modalities of experimental pain. Somatosens Mot Res.

[R12] Esmaeilou Y, Tamaddonfard E, Erfanparast A, Soltanalinejad-Taghiabad F (2022). Behavioral and receptor expression studies on the primary somatosensory
cortex and anterior cingulate cortex oxytocin involvement in modulation of sensory
and affective dimensions of neuropathic pain induced by partial sciatic nerve
ligation in rats. Physiol Behav.

[R13] Kim S, Dowgwillo EA, Kratz AL (2022). Examining the Dynamic Relationship between Positive and Negative Emotions
as a Function of Pain, Fatigue, and Stress in Individuals with and without
Fibromyalgia. The Journal of Pain.

[R14] Feldman SI, Downey G, Schaffer-Neitz R (1999). Pain, negative mood, and perceived support in chronic pain patients: a
daily diary study of people with reflex sympathetic dystrophy syndrome. J Consult Clin Psychol.

[R15] Grouper H, Eisenberg E, Pud D (2021). More insight on the role of personality traits and sensitivity to
experimental pain. J Pain Res.

[R16] Vassend O, Røysamb E, Nielsen CS (2013). Five-factor personality traits and pain sensitivity: a twin study. Pain.

[R17] Banozic A, Miljkovic A, Bras M, Puljak L, Kolcic I, Hayward C, et al (2018). Neuroticism and pain catastrophizing aggravate response to pain in
healthy adults: an experimental study. Korean J Pain.

[R18] Woreta FA, Gupta A, Hochstetler B, Bower KS (2013). Management of post-photorefractive keratectomy pain. Surv Ophthalmol.

[R19] Gadde AK, Srirampur A, Katta KR, Mansoori T, Armah SM (2020). Comparison of single-step transepithelial photorefractive keratectomy and
conventional photorefractive keratectomy in low to high myopic eyes. Indian J Ophthalmol.

[R20] Anisi J (2012). Validity and reliability of NEO Five-Factor Inventory (NEO-FFI) on
university students. International Journal of Behavioral Sciences.

[R21] Mohebbi M, Rafat-Nejad A, Mohammadi SF, Asna-Ashari K, Kasiri M, Heidari-Keshel S, et al (2017). Post-photorefractive keratectomy pain and corneal sub-basal nerve
density. J Ophthalmic Vis Res.

[R22] Mohammadpour M, Khorrami-Nejad M, Shakoor D (2021). Role of artificial tears with and without hyaluronic acid in controlling
ocular discomfort following PRK: a randomized clinical trial. Int J Ophthalmol.

[R23] Baksh BS, Morkin M, Felix E, Karp CL, Galor A (2022). Ocular pain symptoms in individuals with and without a history of
refractive surgery: results from a cross-sectional survey. Cornea.

[R24] Palochak CM, Santamaria J, Justin GA, Apsey DA, Caldwell MC, Steigleman WA, et al (2020). Assessment of factors associated with postoperative pain after
photorefractive keratectomy. Cornea.

[R25] Zarei-Ghanavati S, Nosrat N, Morovatdar N, Abrishami M, Eghbali P (2017). Efficacy of corneal cooling on postoperative pain management after
photorefractive keratectomy: A contralateral eye randomized clinical trial. J Curr Ophthalmol.

[R26] Moradi-Farsani D, Naghibi K, Taheri S, Ali-Kiaii B, Rahimi-Varposhti M (2017). Effects of Age and Gender on Acute Postoperative Pain after Cataract
Surgery under Topical Anesthesia and Sedation. J Isfahan Med Sch.

[R27] Kadu A, Chopra SS, Gupta N, Jayan B, Kochar GD (2015). Effect of the personality traits of the patient on pain perception and
attitude toward orthodontic treatment. J Indian Orthod Soc.

[R28] Abu Alhaija, AlDaikki A, Al-Omairi MK, Al-Khateeb SN (2010). The relationship between personality traits, pain perception and attitude
toward orthodontic treatment. Angle Orthod.

[R29] Tananuvat N, Tansanguan S, Wongpakaran N, Wongpakaran T (2022). Role of neuroticism and perceived stress on quality of life among
patients with dry eye disease. Sci Rep.

[R30] Hanney WJ, Wilson AT, Smith T, Shiley C, Howe J, Kolber MJ (2022). Personality Type and Chronic Pain: The Relationship between Personality
Profile and Chronic Low Back Pain Using Eysenck’s Personality Inventory. NeuroSci.

